# OVX836 a recombinant nucleoprotein vaccine inducing cellular responses and protective efficacy against multiple influenza A subtypes

**DOI:** 10.1038/s41541-019-0098-4

**Published:** 2019-01-23

**Authors:** Judith Del Campo, Andres Pizzorno, Sophia Djebali, Julien Bouley, Marjorie Haller, Jimena Pérez-Vargas, Bruno Lina, Guy Boivin, Marie-Eve Hamelin, Florence Nicolas, Alexandre Le Vert, Yann Leverrier, Manuel Rosa-Calatrava, Jacqueline Marvel, Fergal Hill

**Affiliations:** 1 Osivax, 99, rue de Gerland, 69007 Lyon, France; 2 Virologie et Pathologie Humaine - VirPath Team, Centre International de Recherche en Infectiologie, INSERM U1111, CNRS UMR5308, École Normale Supérieure de Lyon, Université Claude Bernard Lyon 1. Université de Lyon, Lyon, F- 69008 France; 3 Immunity and Cytotoxic Lymphocytes, Centre International de Recherche en Infectiologie, INSERM, U1111, Université Claude Bernard Lyon 1, CNRS, UMR5308, École Normale Supérieure de Lyon. Université de Lyon, F-69007 Lyon, France; 4 Hospices Civils de Lyon, Centre National de Référence des Virus Influenza France Sud, Laboratoire de Virologie, Groupement Hospitalier Nord, Lyon, France; 5 Centre de Recherche en Infectiologie of the Centre Hospitalier Universitaire de Québec and Université Laval, Québec, Canada; 6 Present Address: Enveloped Viruses, Vectors and Immunotherapy Team, Centre International de Recherché en Infectiologie (CIRI), INSERM U1111, Université de Lyon, Lyon, France

## Abstract

Inactivated influenza vaccines (IIVs) lack broad efficacy. Cellular immunity to a conserved internal antigen, the nucleoprotein (NP), has been correlated to protection against pandemic and seasonal influenza and thus could have the potential to broaden vaccine efficacy. We developed OVX836, a recombinant protein vaccine based on an oligomerized NP, which shows increased uptake by dendritic cells and immunogenicity compared with NP. Intramuscular immunization in mice with OVX836 induced strong NP-specific CD4+ and CD8+ T-cell systemic responses and established CD8+ tissue memory T cells in the lung parenchyma. Strikingly, OVX836 protected mice against viral challenge with three different influenza A subtypes, isolated several decades apart and induced a reduction in viral load. When co-administered with IIV, OVX836 was even more effective in reducing lung viral load.

## Influenza vaccine: engineered nucleoprotein with improved protective efficacy against multiple strains

Circulating influenza A virus (IAV) strains differ in their surface proteins each year, and vaccines eliciting an immune response to these proteins are often only partially protective. Internal viral proteins, such as the nucleoprotein (NP), are highly conserved, and cellular immunity to NP has been correlated with protection from diverse strains. However, current IAV vaccines induce a poor immune response to NP. In this study, led by Fergal Hill from Osivax, researchers develop an oligomeric version of NP with improved immunogenicity. Vaccination of mice with oligomeric NP results in an improved NP-specific T-cell response, including CD8^+^ tissue memory T cells in the lung, and protects mice against three different IAV subtypes. Co-administration with the currently used inactivated influenza vaccine further improves protection against virus infection in mice. These results encourage further pre-clinical and clinical development for this vaccine candidate.

## Introduction

Inactivated influenza vaccines (IIVs) aim to prevent infection, and are usually partially successful. A recent meta-analysis from 2004 to 2015 seasons found an efficacy of 33% (confidence interval (CI) = 26–39%) against H3N2 viruses, compared with 61% (CI = 57–65%) against H1N1 and 54% (CI = 46–61%) against influenza B viruses.^1^ During the pH1N1 pandemic of 2009, monovalent vaccines containing an adjuvant had median effectiveness of 69% (CI = 60–93%).^1^ But by targeting only the surface glycoproteins, current influenza vaccines run the risk of having much lower effectiveness should a mismatch occur between the strains included in the vaccine and the strains actually circulating. This happened most recently in the Northern Hemisphere during the 2014–2015 season,^2^ in which vaccine effectiveness was estimated to be as low as 23%,^3^ due to drift mutations in the hemagglutinin antigenic site B of the circulating H3 strain,^4^ but current influenza vaccines retain some effectiveness even when mismatches occur.^5^

Nonetheless, a vaccine approach based on cellular responses to conserved internal antigens could minimize the effects of antigenic drift and even mismatches. Influenza pandemics are very informative in this respect: in a retrospective serological study^6^ of the H2N2 pandemic of 1957, suggestive evidence was found that adults exposed to the new virus were spared from influenza more frequently than children. The author proposed that the main protective factor in adults was immunity resulting from prior infection with earlier (non-H2N2) strains. The recent H7N9 influenza outbreak, with >600 documented cases has been particularly instructive. Indeed, a small study of 16 hospitalized patients lacking neutralizing antibodies showed that those who recovered (12 patients) had significantly stronger CD8+, CD4+, and NK cell immune responses to the H7N9 virus than those (4 patients) who did not recover from the infection.^7^ Noteworthy, early cytotoxic T lymphocyte (CTL) responses were associated with more rapid recovery.^7^

The H1N1 pandemic of 2009 provided further opportunities to determine whether heterosubtypic immunity to influenza exists in humans. A prospective cohort study conducted during the H1N1 pandemic of 2009 showed that individuals who developed less severe illness harbored higher frequencies of pre-existing T cells with specificity for conserved CD8 epitopes.^8^ In human challenge studies, on the other hand, pre-existing CD4+, but not CD8+ T cells responding to influenza internal proteins were associated with lower virus shedding and less severe illness.^9^

Most recently, the Flu Watch Cohort Study^10^ has investigated whether pre-existing T-cell responses targeting internal viral proteins could provide protective immunity against pandemic and seasonal influenza. NP is one of the most conserved internal influenza antigens; even the most divergent protein sequences differ by <11%.^11^ The presence of NP-specific T cells before exposure to virus correlated with less symptomatic, PCR-positive influenza A, during both pandemic and seasonal periods, whereas other conserved antigens were at best of lesser importance.^10^ Additionally, it has been recently possible to demonstrate a strong immune selection against NP CD8+ epitopes in the human influenza lineage when compared with those of the swine lineage. This observation highlights the importance of immune responses against this internal NP antigen, notably considering that other conserved viral antigens, such as the matrix (M1) protein, do not undergo a similar immune selection.^12^

Finally, a comprehensive study comparing systematically the conserved influenza antigens, administered in modified vaccinia virus ankara Modified vaccinia virus ankara (MVA), found that only vectors expressing NP, either alone or in combination with other conserved influenza proteins, protected mice against lethal challenges with H5N1, H7N1, and H9N2.^13^ Altogether, these studies, added to the fact that NP contains CD8+ epitopes that can be dominant in both HLA-A2-positive^14^ and HLA-A2-negative humans,^15^ illustrate the potential utility of cellular immune responses against this conserved NP influenza antigen. NP has been regularly detected in IIV formulations,^16,17^ in variable, but sometimes considerable amounts.^18^ However, it has been shown that, when included in the IIV, NP exerts relatively poor CD8+ T-cell immunogenicity both in humans and in mice.^19^ There is a need, therefore, for novel forms of influenza NP capable of significantly enhancing the immunogenicity of this antigen, even when administered with IIV.

Oligomeric forms of protein antigens have been shown to be more immunogenic than the monomeric forms.^20^ To enhance the immunogenicity of NP, we have developed OVX313, an improved version of the oligomerization domain IMX313^21–26^ created as a hybrid of the C-terminal fragments of two avian C4bp α chain sequences that naturally oligomerize into heptamers.^22^ The heptameric oligomerization domain OVX313 is stabilized by intermolecular disulfide bonds and the extremely high oligomer stability is an interesting scaffold for applications in the field of protein engineering and vaccines.

This study describes OVX836, a recombinant protein candidate vaccine, which consists of the fusion of OVX313 with the nucleoprotein (NP) of influenza A (H1N1/WSN/1933). OVX836 increased immunogenicity over NP and protected against multiple influenza subtypes.

## Results

### Design of OVX836, based on NP fused to a small oligomerization domain and DNA-based immunization

**Fig. 1:**
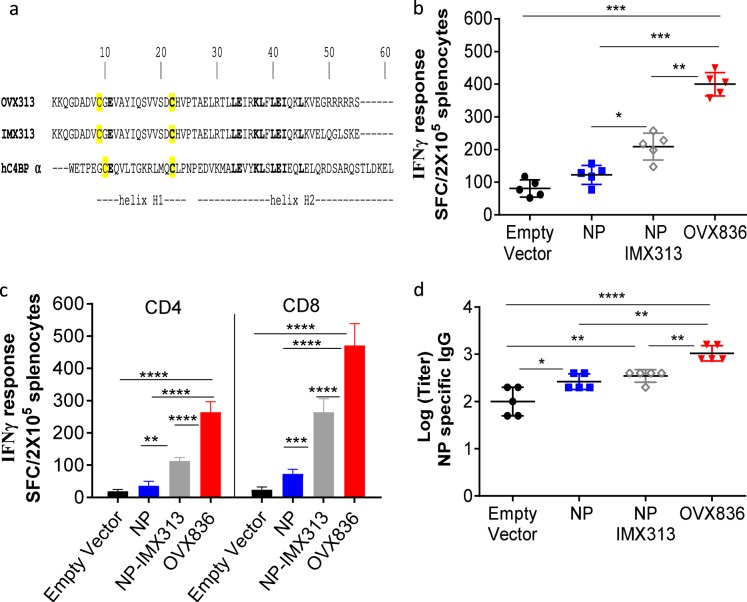
DNA-based immunization with nucleoprotein (NP) fused to a small oligomerization domain. **a** Primary structure of OVX313 oligomerization domain and alignment with IMX313 and the homologous human C4BP-alpha. Residues in bold type are identical. Cysteine residues are highlighted in yellow. **b** C57BL/6 mice were immunized with DNA plasmid encoding NP, NP-IMX313, OVX836 (NP-OVX313), or the empty vector by the intramuscular route. NP-specific interferon-γ (IFN-γ)-secreting cells were evaluated by ELISpot in splenic cells. The results represent the mean (line) and individual data points of each of the five mice with standard deviation of the mean (SD). **c** IFN-γ-secreting CD4+ and CD8+ T-cell, in the splenocytes stimulated with 5 µg/ml of NP protein or 5 µg/ml of NP_366-374_, respectively. **d** Serum anti-NP immunoglobulin G (IgG) were determined using enzyme-linked immunosorbent assay (ELISA). Sera were individually tested in serial dilutions against purified NP protein. The mean (line) of each group is represented with SD. Levels of IgG are expressed as Log (endpoint dilution titer). Differences were assessed by one-way analysis of variance (ANOVA) followed by Tukey’s multiple comparison test with 95% confidence interval, *p* < 0.05 is considered significant. The asterisks refer to the level of significance (*p < 0.05, **p < 0.01, ***p < 0.001, and ****p < 0.0001). Results from a representative experiment of two performed are shown

We set out to make a more immunogenic version of NP by fusing it to the small oligomerization domain OVX313, derived from the previously described IMX313^21,22^ and described in Methods (Fig. 1a). Then, to determine whether the immunogenicity of the influenza NP could be improved by fusing it to either IMX313 or OVX313, four plasmids were constructed (NP, NP-IMX313, NP-OVX313 = OVX836, and empty vector) and used to immunize mice.

Cellular immune responses induced 2 weeks after the last DNA vaccination were measured in spleen by ELISpots using either NP to stimulate total T cells (Fig. 1b) and purified CD4+ T cells (Fig. 1c), or the immunodominant peptide NP_366–374_ to stimulate CD8+ T cells (Fig. 1c). The administration of NP DNA alone induced a weak interferon-γ (IFN-γ)-producing T cells response, not significantly different from the empty vector. On the contrary, we showed that significant amounts of NP-specific IFN-γ-producing T cells, including NP-specific CD4+ and CD8+ T cells response, were induced by OVX836 DNA vaccination, the level of response being increased compared with the response achieved after empty vector, NP or NP-IMX313 DNA vaccination (*p* < 0.01).

In parallel, serum antibody responses to NP were measured 14 days after the last immunization. Mice immunized with NP alone showed only low levels of NP-specific immunoglobulin G (IgG) antibody responses, whereas OVX836 immunized mice had significantly higher IgG antibody responses compared with all other groups of immunized mice (*p* < 0.01) (Fig. 1d).

In summary, our results show that fusing NP to the OVX313 domain improves both NP antigen-specific T-cell and IgG antibody responses, with OVX836 showing immunogenicity superior to NP-IMX313, which is already more immunogenic than NP.

### OVX836 protein is an oligomeric form of NP with improved immunogenicity

**Fig. 2:**
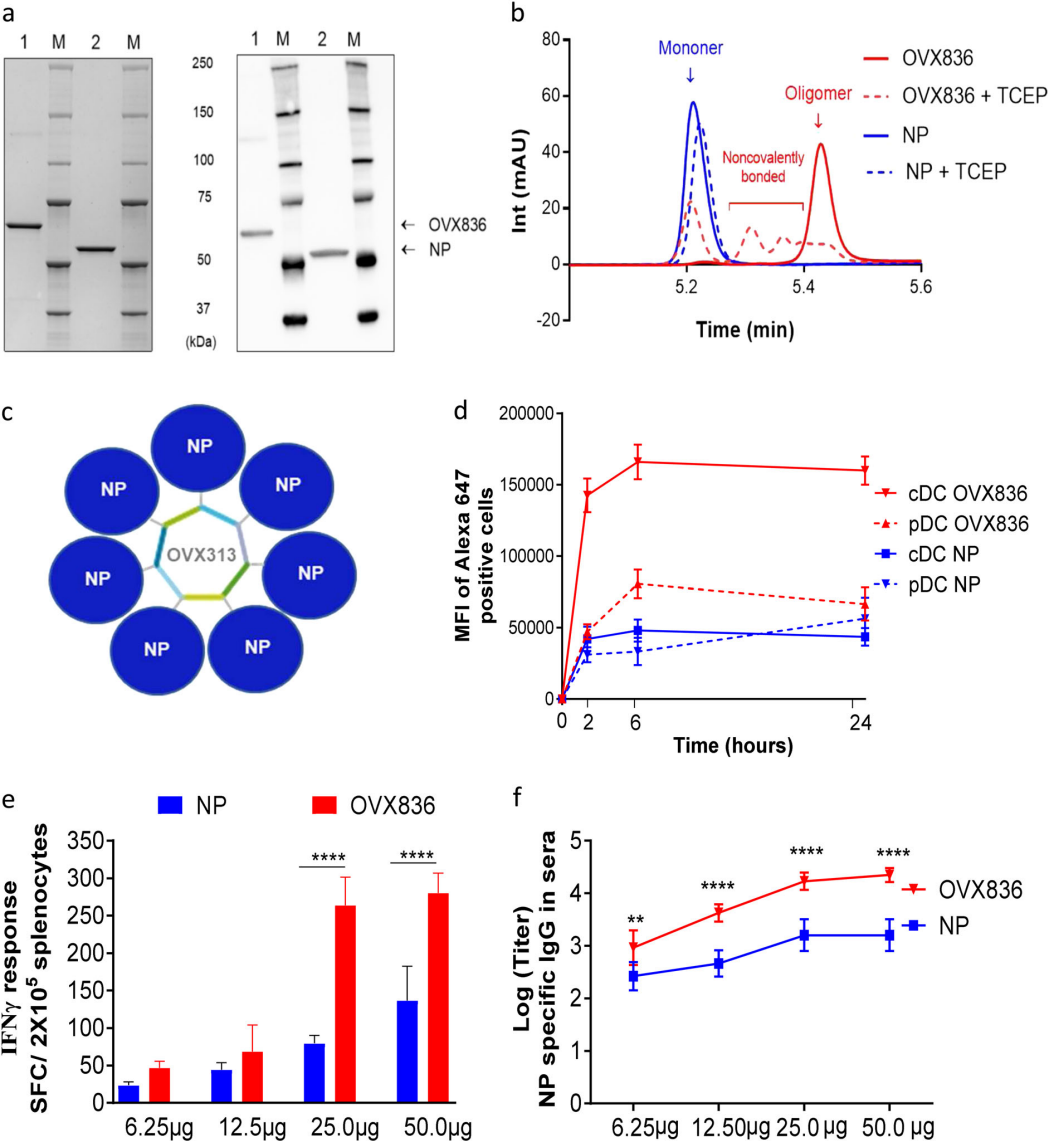
OVX836 protein is an oligomeric form of nucleoprotein (NP) with improved immunogenicity. **a** Representative SDS-PAGE analysis of OVX836 and NP proteins. Polypeptides were fractionated on 4–12 % Bis-tris gels and stained with Instant Blue, total protein were stained (left panel) or blotted onto a membrane and probed with anti-NP Mab (right panel). OVX836 and NP migrated at 64 and 56 kDa, respectively (lanes 1 and 2). Molecular weight (MW) markers (lanes M). The SDS-PAGE and the western blot derive from the same experiment and were processed in the same day. **b** Recombinant protein species were separated by reversed-phase (RP) liquid chromatography: the R*t* of ∼5.45 min (red chromatogram) corresponds to the oligomeric structure of OVX836, and the R*t* of ∼5.22 min to the monomer of NP. Noncovalently bonded subunits, most likely dimers, trimers, and tetramers of NP, were chromatographically separated from oligomeric OVX836 following reduction (red dashed line). Intensity (Int). **c** Schematic representation of OVX836 protein heptamer. **d** Kinetics of incorporation of OVX836-Alexa647 by Flt3L-DC Bone marrow. Dendritic cells (DCs) were incubated for 2, 6, or 24 h with OVX836-Alexa647 or NP-Alexa647 (50 µg/ml) at 37 °C and surface stained for CD11c and B220. Alexa647 MFI of cDC (CD11c+, B220-) and pDC (CD11c+, B220+) populations were measured. Data show means ± SD. of two independent experiments. **e** Comparison of NP-specific interferon-γ (IFN-γ)-producing cells evaluated by ELISpot in total splenic cells (C57BL/6 mice immunized twice, intramuscularly, with 6.25, 12.5, 25, and 50 µg of NP or OVX836). Results show number of IFN-γ-secreting cells per 2 × 10^5^ cells per well. **f** NP-specific immunoglobulin G (IgG) were measured by enzyme-linked immunosorbent assay (ELISA) in serum from the same C57BL/6 mice groups. Levels of IgG are expressed as Log (endpoint dilution titer). The results represent the mean (line) and individual data points of each of the five mice with SD. Differences were assessed by one-way analysis of variance (ANOVA) followed by Tukey’s Multiple comparison test with 95% confidence intervals; *p* < 0.05 is considered significant (****p < 0.0001) or by two-way ANOVA followed by a Dunnett’s multiple comparison test (multiple time points) (**p *<* 0.01; ****p *<* 0.0001). These experiments were performed three times with similar results

Based on the results obtained with DNA immunizations, two recombinant proteins were produced in *Escherichia coli*: NP from the prototype human isolate A/Wilson-Smith/1933 (ref. ^27^), referred to here as NP, and the same antigen fused through its C-terminus to OVX313, called OVX836. Both OVX836 and NP proteins were purified using heparin sepharose affinity columns. The purity of the recombinant proteins was analyzed by Sodium dodecyl sulfate polyacrylamide gel electrophoresis (SDS-PAGE) under reducing and denaturing conditions (Fig. 2a, left). OVX836 and NP resolved as single bands at approximatively 64 and 56 kDa, as expected from the cloned amino-acid sequences (theoretical MWs of 62.7 and 56.2 kDa, respectively). Moreover, OVX836 and NP were recognized by a Monoclonal antibody (MAb) raised against NP (Fig. 2a, right).

OVX836 and NP were then examined by means of reversed-phase (RP) chromatography, showing that purified recombinant proteins were very homogeneous (Fig. 2b). Both molecules exhibited a single-elution peak that represents >97% of the total protein area at 280 nm. OVX836 and NP showed a main peak corresponding to the heptameric and monomeric form of NP, respectively. As expected, treatment with the reducing agent tris(2-carboxyethyl)phosphine (TCEP) did not modify the chromatographic profile of NP. In contrast, the reduction of disulfide bonds promoted dissociation of the OVX836 subunits into lower association states (Fig. 2b), showing that intermolecular disulfide bonds were required for the stabilization of the heptameric state of OVX836 under RP conditions. Under aqueous conditions, OVX836 is heptameric independently from disulphide bonds.

Based on the results from gel filtration chromatography, the most abundant form of NP was a trimer of 150 kDa and only few monomers were observed (Supplementary Figure 1a). The analysis of the oligomeric state of purified recombinant NP has indicated a broad distribution of oligomeric states.^28,29^ In contrast, a mutated version of NP did not self-associate and formed only monomers of 55 kDa as the E339A and R416A mutations in the tail loop result in the complete loss of NP trimerization.^29^ The heptameric state of OVX836, as well as small oligomers of heptamers (di-/tri-heptamers) were consistently observed (MW in a range from 400 to 1200 kDa), which is consistent from batch-to-batch. Interestingly, the OVX836 tail loop mutant did not show small oligomers of heptamers (Supplementary Figure 1b).

Therefore, OVX836 and NP are pure and homogenous proteins, and compared with the native NP trimer of 150 kDa, OVX836 is mainly organized as mono-, di-, and tri-heptamers under aqueous conditions (400–1200 kDa) (Supplementary Figure 1a). Our experiments also indicated that OVX836 is a stable heptamer due to noncovalent intermolecular interactions, as well as the formation of disulfide bridges between the subunits. The Fig. 2c shows a schematic representation of OVX836 protein heptamer.

We then investigated the mechanisms by which OVX836 could be up-taken by professional antigen-presenting cells such as dendritic cells (DCs). DCs were generated in vitro from murine bone marrow cultivated with Flt3 ligand (Flt3L), as a convenient source of pDC and cDC that are equivalent to the spleen CD8α+ and CD8α– DC.^30^ We first checked whether OVX836 was internalized by the different DCs subset. Imaging using ImageStreamX showed a strong internalization of OVX836, localizing in vesicles within DCs (Supplementary Figure 2a, b). Kinetic studies using flow cytometry using Alexa647-labeled proteins (3 mol dye/mol protein) confirmed that OVX836 and NP products are rapidly internalized (within 2 h) by DC (Fig. 2d). The levels of incorporation are dose and time dependent (percentage and MFI), with OVX836 being more efficiently incorporated than NP, even after 24-h incubation.

The immunogenicity of the recombinant proteins NP and OVX836 were measured in C57BL6 mice following two intramuscular (i.m.) injections with doubling doses from 6.25 µg to 50 µg. Fourteen days after the last immunization, immune responses against the NP protein were detected by ex vivo IFN-γ ELISPOT to measure NP-specific IFN-γ-producing T cells in response to NP stimulation (Fig. 2e). The results showed that the NP induced moderate T-cell immune responses and OVX836 protein was more immunogenic at all doses than NP, reaching a maximum response from 25 µg (*p* < 0.0001).

Furthermore, OVX836 elicited high titer serum NP-specific IgG antibodies, which increased with the dose and were higher than with NP at all doses (*p* < 0.01) (Fig. 2f). Together, these results showed that the fusion protein OVX836 more effectively stimulates T cells and B cells than NP alone.

### OVX836 induces high NP-specific systemic immunogenic responses, local T cells in the lung, and can be used in combination with IIV

**Fig. 3:**
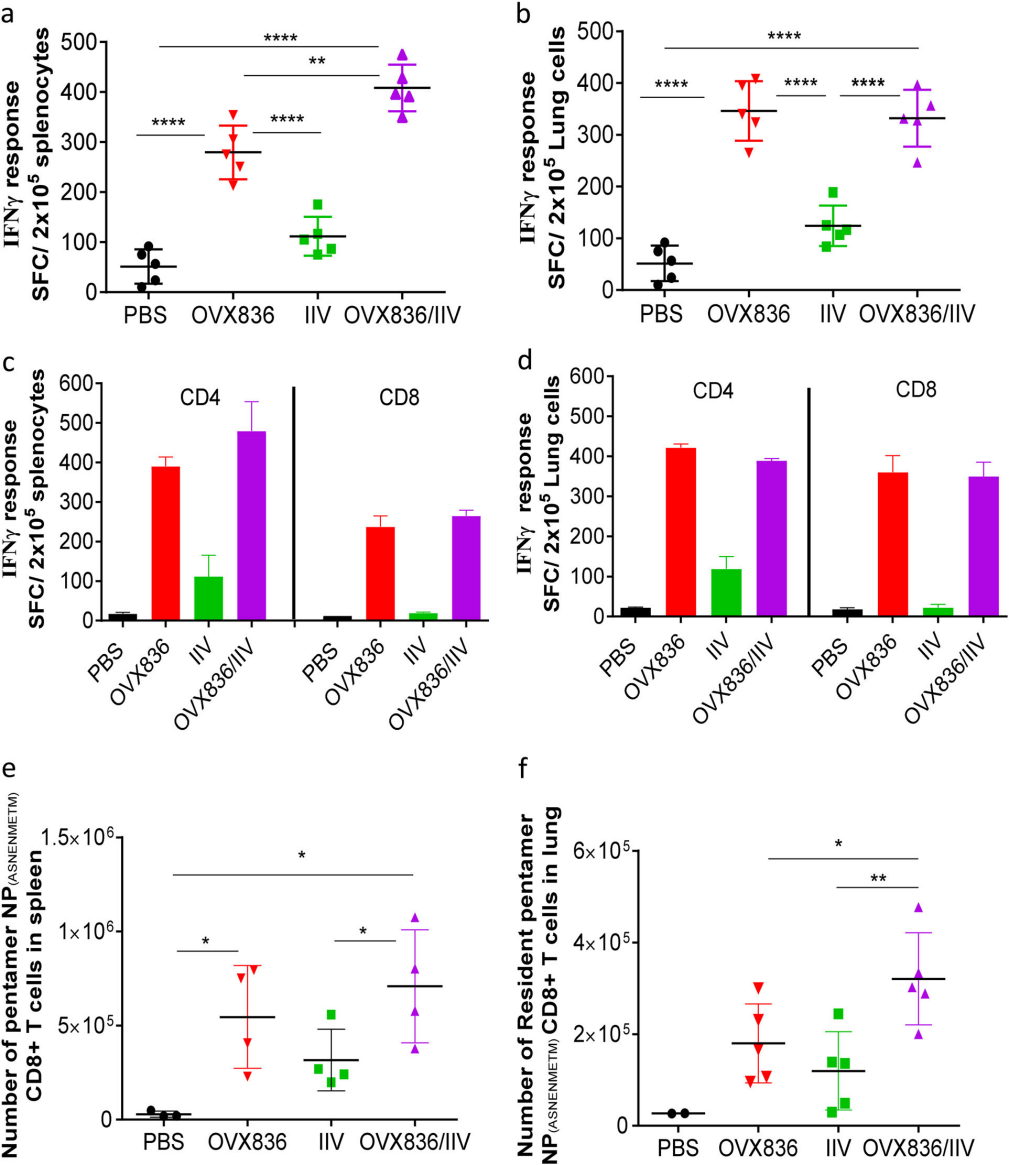
OVX836 vaccine alone or combined with inactivated seasonal influenza vaccine induced humoral and cellular responses in the periphery and in the lung. C57BL/6 mice were immunized twice, intramuscularly with 25 µg of OVX836; with 50 µL of IIV (1.5 µg of each HA); or with both combined (OVX836/IIV), by injection into two different muscles in the same hind limb. A group injected with PBS was used as control. **a**, **b** cellular immunogencity following immunization in mice. Nucleoprotein (NP)-specific interferon-γ (IFN-γ)-secreting T cells (spot-forming cells (SFC)) evaluated by ELISPOT per 2 × 10^5^ cells in the spleen and in the lung compartments. Results are shown for three independent experiments. The results represent the mean (line) and individual data points of each of the five mice. **c**, **d** Comparison of specific IFN-γ-secreting CD4+ and CD8+ T-cell evaluated by ELISPOT in the spleen or in the lung compartments. **e**, **f** NP-specific CD8+ T cells in the spleen and lung compartments were detected by flow cytometry using pentamer staining (H2Db-ASNENMETM). **e** Number of Pentamer CD8+ T cells in the spleen and **f** in the lung. Lung parenchyma (resident) cells were identified using anti-CD45 intravascular staining. Data are representative of three independent experiments (mean of five mice per group). Differences were assessing by one-way analysis of variance (ANOVA) followed by Tukey’s Multiple comparison test with 95% confidence intervals; *p* < 0.05 is considered significant (*p < 0.05, **p < 0.01, ****p < 0.0001)

We next studied the effect of OVX836 at 25 µg alone, or in association with the IIV in C57BL/6 mice, following two i.m. injections; these co-administrations took place in the same hind limb but with injection into different muscles. To characterize the cellular immune responses elicited in mice, NP-specific IFN-γ after the two immunizations were quantified using ELISPOT assays. In the group immunized with OVX836 or OVX836 combined with IIV, a strong NP-specific IFN-γ-producing T cells response was induced in the spleen and in lung. This response was significantly higher than in control group *p* < 0.0001 (Fig. 3a, b). IIV as expected elicited only low levels of NP-specific T-cell responses. In addition, in both the spleen and lung, OVX836 and the combination of OVX836 with IIV by the i.m. route were highly immunogenic, inducing NP-specific CD4+ and NP_366-374_-specific CD8+ T cells response >10-fold higher than phosphate buffered saline (PBS) group (Fig. 3c, d).

To additionally characterize the NP-specific immune response induced by OVX836, we assessed the magnitude of cytokines released in the culture supernatants from the lung cells stimulated with NP_366-374_ peptide, using an enzyme-linked immunosorbent assay (ELISA) (Mabtech). Immunization with OVX836 alone or in combination with IIV induced significantly higher levels of IFN-γ, tumor necrosis factor-α (TNF-α), and interleukin-2 (IL-2) compared with control PBS or IIV group (but not IL-4) (Supplementary Figure 3). These data suggest that OVX836 was able to induce a strong Th1 immune response in the lung with significant increases in the production of IFN-γ, TNF-α, and IL-2 cytokines that can contribute to influenza clearance.

Specific CD8+ T-cell response in the lung and spleen by ELISpot, was induced by OVX836, suggesting that the antigen was cross presented. We also analyzed the quality of the CD8+ T cells induced by OVX836 alone or in combination with IIV. The frequency of NP_366-374_-specific CD8+ T cells following immunization was assessed using pentamer staining by flow cytometry (Fig. 3e, f) 14 days after the last immunization to confirm the CD8+ T-cell activation. Within the spleens of mice, we observed an increased percentage of NP_366-374_-specific CD8+ T cells following immunization with OVX836 alone or in combination with IIV compared with IIV or to unvaccinated groups (Fig. 3e). We next analyzed the presence of NP_366-374_-specific memory CD8+ T cells within the lung parenchyma using intravascular staining to exclude cells in the lung vasculature. Immunization with OVX836 induced the generation of NP_366-374_-specific memory CD8+ T cells that were located within the lung parenchyma (Fig. 3f). Interestingly, the combination of OVX836 with IIV elicited even higher numbers of lung resident memory CD8+ T cells (*p* < 0.05). These results indicate that OVX836 induced virus-specific memory CD8+ T cells that can infiltrate lung tissues.

Taken together, our results show that OVX836 induces strong systemic NP-specific CD4+ and CD8+ T cells response, as well as NP-specific IgG; it also induces lung memory CD8+ T cells against NP localized in the lung parenchyma.

### Vaccination with OVX836 protects mice against lethal infection with the influenza H1N1 A/WSN/33 strain

**Fig. 4:**
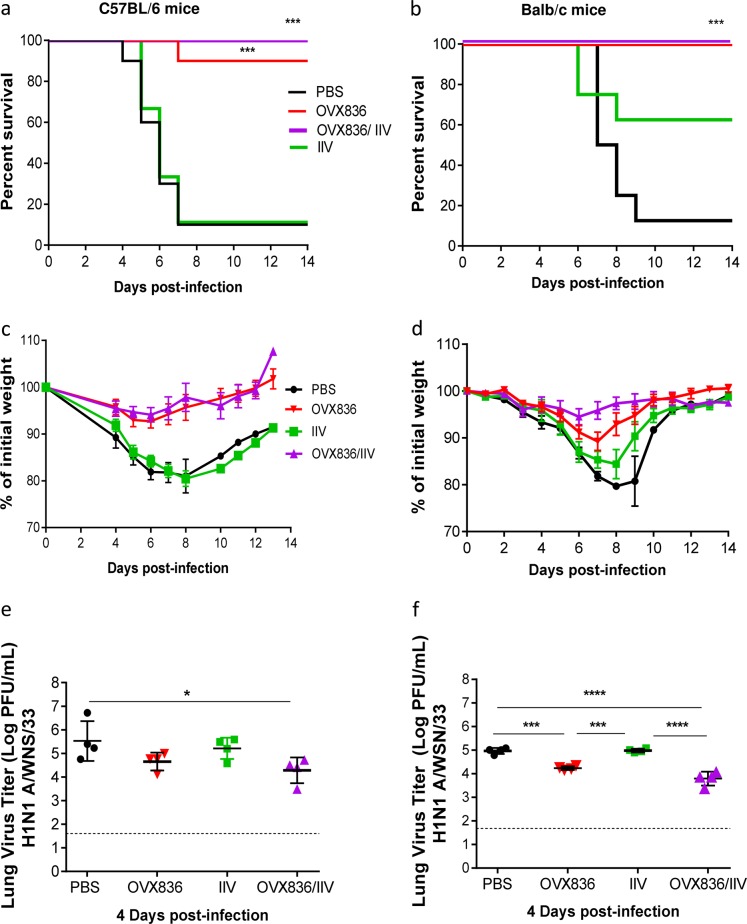
Vaccination with OVX836 protects mice against lethal infection with influenza H1N1 A/WSN/33 strain. Groups of 12 female C57BL/6 (a, **c**, **e**) or Balb/c mice (**b**, **d**, **e**) received two 21 days apart intramuscular (i.m.) immunizations with either PBS, IIV, OVX836, or a combination of IIV and OVX836 (OVX836/IIV). Three weeks after the second immunization, mice were intranasally (i.n.) infected with 2LD50 (C57BL/6) or 1LD50 (Balb/c) of H1N1 A/WSN/33 virus. **a**, **b** Percent survival rates. *p < 0.05, **p < 0.01 and ***p < 0.001 by log-rank (Mantel-Cox) or Kruskal–Wallis (Dunn’s multiple comparison) tests. **c**, **d** Mean weight changes (±SEM), **e**, **f** mean lung viral titers expressed as log (PFU/mL ± SD) on day 4 post-infection. Individual virus titers are given and lines represent averages. In all, 1.7 log PFU/mL is the detection limit of the technique (dotted line). Statistical significance of the variance between multiple groups was calculated with Kruskal–Wallis test followed by Dunn’s multiple comparison test (*p < 0.05, ***p < 0.001, ****p < 0.0001)

The most important feature of an effective vaccine candidate is the capacity to protect individuals against symptoms and signs of infection. To evaluate this aspect, we performed experimental infections in C57BL/6 mice previously immunized with either PBS (control), IIV, OVX836, or a combination of IIV and OVX836 (OVX836/IIV), using the H1N1 A/WSN/33 influenza virus, harboring the same NP protein present in OVX836. In order to eliminate possible model-associated bias in the interpretation of results and anticipating further pre-clinical evaluation, identical experiments were also performed using the other classic influenza infection mouse model, Balb/c mice. As shown in Fig. 4, intranasal (i.n.) inoculation of PBS-immunized (control) mice with H1N1 A/WSN/33 influenza strain resulted in 90% mortality and 20% maximum mean weight loss in both mouse strains. On the other hand, whereas immunization with IIV conferred partial protection (60% survival rates) against the H1N1 A/WSN/33 challenge (not included in the IIV formulation) in Balb/c, it failed to protect C57BL/6 mice (10% survival rate). More importantly, both OVX836 and OVX836/IIV vaccinated groups were highly protected against viral challenge in both mouse strains, as shown by survival rates of 90 and 100% in C57BL/6 and 100 and 100% in Balb/c mice, respectively (Fig. 4a, b). Additionally, although a mild weight loss was observed in Balb/c mice immunized with OVX836 (10% maximum mean weight loss (Fig. 4d)), no significant weight loss (<5%) was observed for either the OVX836 or OVX836/IIV immunized in C57BL/6 (Fig. 4c). OVX836 and OVX836/IIV vaccination also induced 1-log or higher reductions in lung viral titers (LVTs) compared with the PBS control group, especially in the case of the OX836/IIV combination (Fig. 4e, f). Finally, both pre- and post-challenge hemagglutination inhibition (HAI) titers against the H1N1 A/WSN/33 virus, as well as the viral strains contained in the IIV formulation were comparable between the two mouse strains, hence validating the IgG responses expected as a result of both IIV vaccination and challenge (Table 1). Of note, OVX836 vaccination did not induce any HAI responses, and tended to increase the HAI response toward some viral strains included in the IIV.

**Table 1:** Hemagglutination inhibition titers

			Viral strain used for HAI assay							
			A/WSN/33 ^b^		A/California/2009		A/Texas/54		B/Massachusetts	
			Pre	Post	Pre	Post	Pre	Post	Pre	Post
			Challenge		Challenge		Challenge		Challenge	
C57BL/6 mice	PBS	Pool 1	<10		<10		<10		<10	
		Pool 2	<10	80^a^	<10	<10^a^	<10	<10^a^	<10	<10^a^
		Pool 3	<10		<10		<10		<10	
	OVX836	Pool 1	<10	320	<10	<10	<10	<10	<10	<10
		Pool 2	<10	320	<10	<10	<10	<10	<10	<10
		Pool 3	<10	320	<10	<10	10	<10	<10	<10
	IIV	Pool 1	<10		20		80		10	
		Pool 2	<10	160^a^	20	20^a^	40	40^a^	10	10^a^
		Pool 3	<10		10		80		<10	
	OVX836/IIV	Pool 1	20	160	80	80	40	20	20	10
		Pool 2	<10	160	40	40	40	40	20	20
		Pool 3	20	160	80	80	40	20	20	10
Balb/c mice	PBS	Pool 1	<10		<10		<10		<10	
		Pool 2	<10	160^a^	<10	<10^a^	<10	<10^a^	<10	<10^a^
		Pool 3	<10		<10		<10		<10	
	OVX836	Pool 1	<10	160	<10	<10	<10	<10	<10	<10
		Pool 2	<10	80	<10	<10	<10	<10	<10	<10
		Pool 3	<10	160	<10	<10	<10	<10	<10	<10
	IIV	Pool 1	<10	160	20	80	160	320	10	20
		Pool 2	<10	160	20	80	160	160	10	10
		Pool 3	<10	80	10	40	80	80	10	10
	OVX836/IIV	Pool 1	<10	80	40	40	40	40	20	10
		Pool 2	<10	160	40	40	80	80	10	10
		Pool 3	10	160	40	40	160	320	10	20

Serologic tests against the virus strain used for the viral challenge, as well as the strains contained in the IIV vaccine

HAI titers were measured in pooled serum samples (three pools per group) and expressed as the reciprocal of the limit dilution. Serologic tests were performed against the same virus strain used for the viral challenge (H1N1 A/WSN/33 virus), as well as the strains contained in the IIV vaccine (A/California/7/2009 (H1N1), A/Texas/50/2012, and B/Massachusetts/2/2012)

*HAI* hemagglutination inhibition

^a^Results from one remaining mouse

^b^Strain used for viral challenge

These results show that OVX836 immunization protected both C57BL/6 and Balb/c mice against a lethal challenge infection with H1N1 A/WSN/33 strain. Moreover, when IIV and OVX836 were co-administered, decreased viral load in lungs were observed.

### Vaccination with OVX836 induces broad protection against lethal challenge with different influenza A subtypes

**Fig. 5:**
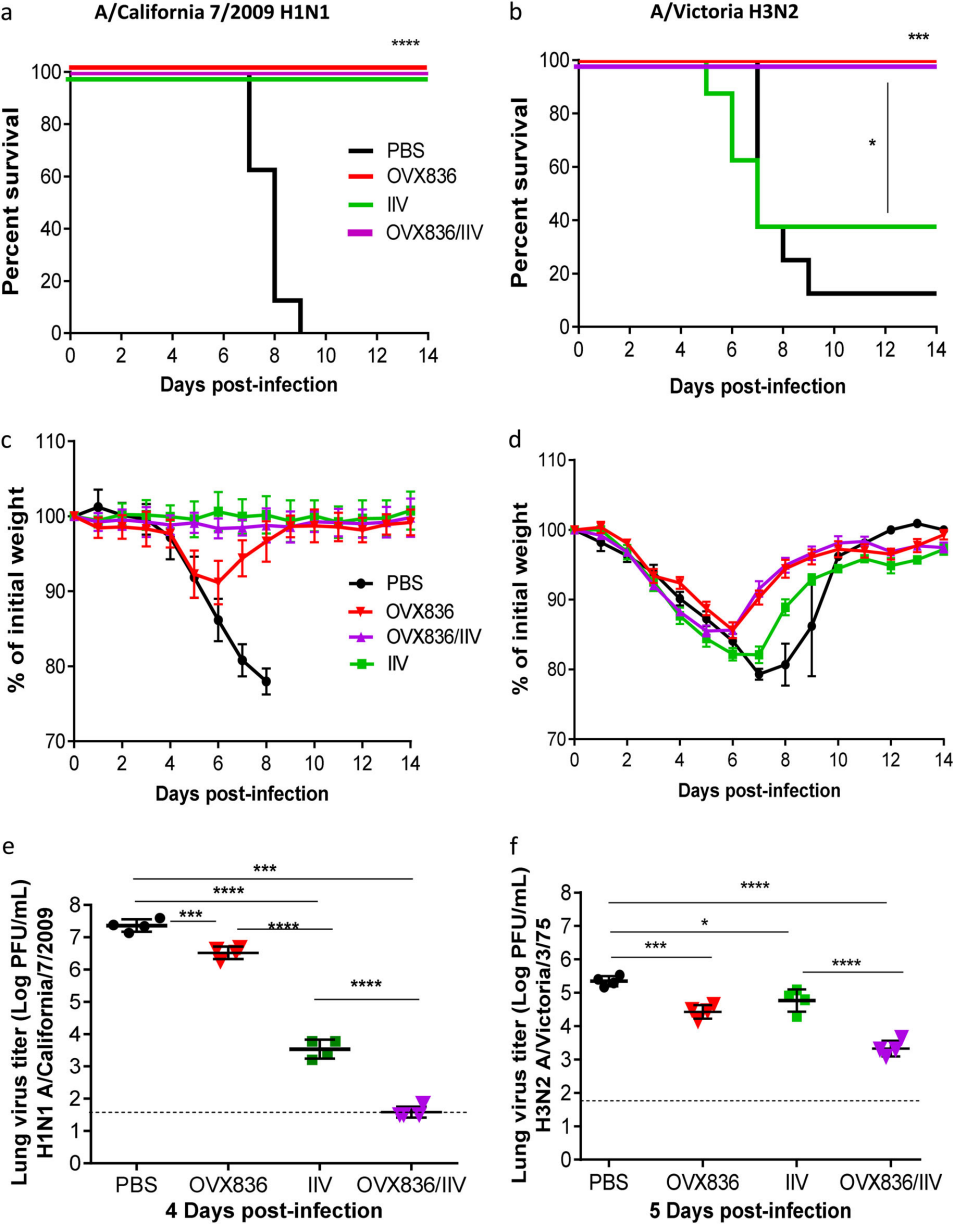
Vaccination with OVX836 induces broad protection against lethal challenge with different influenza A subtypes. Groups of 12 female Balb/c mice received two 21 days apart intramuscular (i.m.) immunizations with either PBS, inactivated influenza vaccine (IIV), OVX836, or a combination of IIV and OVX836 (OVX836/IIV). Three weeks after the second immunization, mice were intranasally (i.n.) infected with either 3LD50 of influenza H1N1 A/California/7/2009 (left panel) or 1LD50 of H3N2 A/Victoria/5/72 (right panel) virus. **a**, **b** Percent survival rates. *p < 0.05, **p < 0.01 and ***p < 0.001 by log-rank (Mantel–Cox), **c**, **d** mean weight changes (±SEM), **e**, **f** mean lung viral titers expressed as log (PFU/mL ± SD) on day 4 or 5 post-infection. Individual virus titers are given and lines represent averages. In all, 1.7 log PFU/mL is the detection limit of the technique (dotted line). Statistical significance of the variance between multiple groups was calculated with Kruskal–Wallis test followed by Dunn’s multiple comparison test (*p < 0.05, ***p < 0.001,****p < 0.0001)

In view of the positive results obtained for H1N1 A/WSN/33 infection in two different mouse strains, we further investigated the protective capacity of OVX836 against two additional influenza subtypes A, in the Balb/c lethal infection model (Fig. 5). Mice were immunized with either PBS, IIV, OVX836, or a combination of IIV and OVX836 (OVX836/IIV) following the same experimental protocol described above, and then i.n. infected with either H1N1 A/California/7/2009 (homologous to the H1N1 strain present in the IIV and containing an NP protein with 93% identity to the OVX836 NP protein) or H3N2 A/Victoria/3/75 (heterologous to IIV and containing an NP protein with 90% identity to the OVX836 NP protein) viruses. Of note, preliminary experiments were performed in order to calibrate the viral inoculum and lung sampling time-point to obtain comparable mortality rates among the different viral strains used, as well as to measure LVTs at the peak of viral replication. Infection of PBS-immunized (control) mice with H1N1 A/California/7/2009 or H3N2 A/Victoria/3/75 resulted in 100 and 90% mortality, respectively, as well as >20% maximum mean weight loss in both cases (Fig. 5a, b). As expected, the two IIV-containing groups (IIV and OVX836/IIV) showed no signs of mortality or weight loss following infection with the H1N1 A/California/7/2009 virus, which in turn produced mild (>10%) weight loss in the OVX836 group with 100% survival rate (Fig. 5a, c). This observation is further supported by 1-log, 4-log, and 6-log reductions in mean LVTs for the OVX836, IIV and OVX836/IIV groups, respectively, compared with the PBS control group (Fig. 5e).

IIV conferred only partial protection (40% survival and 18% weight loss) against the non-homologous H3N2 A/Victoria/3/75 virus. Conversely, both OVX836 and OVX836/IIV vaccinated groups were fully protected (100% survival) against lethal viral challenge with the H3N2 influenza A strain, yet showing 15% mean maximum weight losses. Indeed, the main difference for these two groups in terms of weight loss was driven by a shorter recovery time compared with the PBS and IIV groups (Fig. 5b, d). OVX836 and OVX836/IIV vaccination induced 1-log and 2-log reductions in mean LVTs, respectively (Fig. 5f). Once again, HAI titers against the viral strains used for infection, as well those included in the IIV vaccine formulation confirm the expected antibodies response following IIV vaccinations and challenge (Supplementary Table 1). Interestingly, pre-challenge H1N1 A/California/7/2009 HA titers induced by the OVX836/IIV group tended to be higher than those of the IIV group alone and OVX836 vaccination did not induce any HAI responses, consistent with the observations of Table 1.

Taken together, the results of the different challenge tests demonstrate the efficacy of OVX836 alone to provide protection against lethal viral infection with different influenza A strains. In addition, when OVX836 and IIV were combined for the vaccination, IIV being either homologous or heterologous to the viral strain used for the challenge, decrease of the viral loads in lungs were achieved.

## Discussion

Influenza is one of the major infectious disease threats for humans in terms of annual health impact of seasonal influenza and in terms of potential dramatic global consequences of an influenza pandemic outbreak.^31^ Licensed influenza vaccines induce antibodies against the hemagglutinin, a highly polymorphic surface antigen, and as such, need to be reformulated and re-administered every year. A vaccine providing protective immunity against highly conserved, mostly internal, antigens could provide longer-lasting protection and cover multiple influenza subtypes.^32,33^ However, even though such universal influenza vaccines are subject to much discussions and many reviews, only a handful of candidates have reached the clinical testing stage.^34,35^ Despite their limited efficacy,^34^ current seasonal vaccines remain the most effective way to prevent influenza outbreaks and annual vaccination is recommended for all people aged ≥6 months in the United States and Europe.^36–38^

A wide consensus developed the hypothesis that NP probably plays a role in influenza protection with the induction of NP-specific CD8+ T-cell responses mainly through CTL cross-reactions.^19,39–43^ B cells activation and NP antibody response could also play a role in the protection against multiple influenza A strains.^19,44^ Since the pioneering studies of Wraith and his colleagues in the 1980s, it has been shown that the influenza NP purified from virions can induce good but incomplete protection against homologous and heterologous viral challenges in mice.^45^ The same group had previously shown that a combination of NP and HA primed the development of cytotoxic T cells that were cross-reactive against other influenza A viruses (IAVs).^46^

Furthermore, several research groups reported the capacity of different NP-based vaccines to accelerate viral clearance and prevent mortality in mice subsequently challenged with either matched or mis-matched IAV strains.^47^ In that regard, multiple studies on NP-based vaccines in the form of recombinant proteins,^47–51^ DNA vaccines,^40–42,45,46^ or viral vectors,^13,52–56^ confirmed the protective role of CTL immune responses targeting NP.

In this context, we developed a more immunogenic version of NP, capable of eliciting stronger and protective cytotoxic T-cell responses at systemic and mucosal site (lung). We initially fused NP to our first-generation oligomerization domain IMX313,^21^ and then to its improved version OVX313. Comparing with the IMX313, the C-terminal part of the OVX313, includes a short arginine cationic sequence to facilitate vaccine purification through chromatography. Through a DNA-based vaccine study, we compared the level of immunogenicity generated by the “naked” NP antigen and of NP fused to our technology, and found that the fusion to IMX313 increased the immunogenicity of the NP antigen, which was further significantly boosted by fusion to OVX313. Leveraging these DNA vaccine results, we developed a recombinant protein vaccine harboring the NP protein from the prototype influenza H1N1 A/WSN/33 human strain^27^ fused to OVX313. Our technology platform (OVX313) enables the production of an heptamerized form of the NP antigen, an OVX836 recombinant protein vaccine candidate. Several studies demonstrated that oligomerization of antigens using this platform (through OVX313 tag or its previous version IMX313) improves humoral and cellular immunogenicity in animals (Malaria antigens,^23^ Tuberculosis antigen 85A,^24^ Malaria Pfs25 antigen,^25^ HPV L2 antigen^26^).

OVX836 enhanced antigen uptake by DCs, and induced more NP-specific CD4+ and CD8+ T cells response, as well as NP-specific IgG compared with NP alone. When we compared OVX836 alone, in combination with an IIVs and IIV alone in a mice immunization study, we showed that OVX836 generated significantly higher T-cell responses than IIV alone. The cellular response obtained with the combination of IIV and OVX836 was similar to the one obtained with OVX836 alone. OVX836 induced high frequencies of cross-reactive NP-specific T cells, notably CD8+ T cells that were located in the lung parenchyma. For a long time, i.m. immunization has been considered as unable to generate mucosal immune responses. In the 2016, Su, et al. have published a large review of the literature demonstrating that i.m. vaccination can promote both systemic and mucosal immune responses, and protect against mucosal pathogen challenge.^57^ In our study, we demonstrated that i.m. immunization with OVX836 enhances NP-specific T-cell response localized in the lung parenchyma as shown by pentamer analysis using CD45 intravascular staining method.

Mice challenge studies showed that OVX836 alone provides effective protection against lethal infection with different subtypes of influenza A (A/WSN/33, H1N1, or H3N2). Based on current scientific knowledge, we hypothesize that the protection provided by administration of OVX836 alone is mostly mediated by CD8+ T-cell responses against the widely conserved NP antigen, with a potentiating action of CD4+ T-cell responses, and the potential contribution of NP-specific antibody responses. In the 1980s, Wraith et al. made the hypothesis that immune responses against NP would not prevent infection, but rather accelerate clearance of infected cells.^40,41^

This hypothesis is in line with the weight curves observed for most of the groups immunized with OVX836 alone, which experience initial body weight loss following the viral challenge comparable to that of the PBS-vaccinated mice, but with less severity and significantly faster recovery time. As a result, it shows the potential of OVX836 to provide broad-spectrum protection against moderate to severe cases caused by multiple A-strain viruses. Another attractive immunization strategy could be to leverage two lines of attack by associating NP-targeted CD8+ T-cell response provided by OVX836 with the humoral response against HA generated by the IIV. This is supported by our results showing decrease viral loads in mice, when OVX836 and IIV are used in combination, the IIV being either homologous or heterologous to the viral strain used for the challenge. Such combined vaccination strategy could prevent moderate to several cases cause by all influenza A subtypes and at the same time limit viral dissemination even when antibodies are less effective in neutralizing the virus.

Furthermore, we also noticed, with some viral strains used for the HAI assay, slightly better pre-challenge HAI titers in the OVX836/IIV vaccinated groups compared with those vaccinated with IIV alone. Although the real implications of this relatively mild increase remain to be determined, a potential contribution of OVX836 to the HA-specific humoral response induced by IIV might be worth further study.

The broad annual vaccination recommendation for people aged >6 months with current IIV might represent a double-edged weapon in the long term, since on one side, it confers partial yet important protection against circulating strains, but on the other side, it could create a potential risk that vaccine recipients will not develop the lasting heterosubtypic immunity usually acquired through repeated natural infections.^38^ An immunization strategy with a vaccine generating cellular response against NP, like OVX836, could present a double advantage by protecting against moderate to severe cases while not preventing subject’s natural infection and the development of lasting heterosubtypic immunity. Based on the mice experiments conducted so far, OVX836 is a good candidate vaccine in order to effectively induce cell-mediated immunity to a conserved antigen and provide wide protection against multiple A-strain influenza viruses. As NP is highly conserved with >95% homology within A strains,^58^ OVX836 represents an attractive standalone vaccination strategy to prevent influenza caused by circulating or emerging A strains, especially in a case of a pandemic outbreak. In the context of limited effectiveness of licensed seasonal vaccines, OVX836 immunization strategy could also be combined with current or next-generation vaccines triggering antibody responses against surface antigens in order to provide complete protection against multiple circulating and emerging A and B influenza subtypes.

## Methods

### OVX313, an improved version of the heptamerization domain IMX313

IMX313 is a 55-amino-acid protein domain, obtained as a hybrid of the C-terminal fragments of two avian C4bp proteins that naturally oligomerize into heptamers. Of note, in our IMX313 construction, avian C4bp sequences were engineered in order to minimize identities with their human counterparts. We have previously observed that following its fusion to a monomeric antigen, IMX313 allows the presentation of seven copies of the antigen to the immune system, increasing both B and T-cell immunogenicity.^21,59^ Based on this observation, we designed for the present study OVX313 as an optimized version of IMX313. We improved its oligomerization domain by substituting the last seven amino acids of IMX313 (originally LQGLSKE) by seven different amino acids (GRRRRRS), leaving the total length at 55-amino acids, which retains a heptameric core structure stabilized by monovalent intermolecular interactions and disulfide bonds.

### Construction of plasmids

Four plasmids were constructed for DNA immunizations by modifying pGT-h-NP, a plasmid expressing NP,^60^ to create four new ones. First, the human tPA signal peptide sequence was amplified and inserted in-frame with the N-terminus of the NP coding sequence in pGT-h-NP creating a eukaryotic expression plasmid (pIMX714) for secreted NP,^59^ using the forward primer IMX1305 (5′-CACTGAGTGACATCAAAATCATGGATGCAATGAAGAGAGGGC-3′) and the reverse primer IMX1306 (5′-CGTAAGACCGTTTGGTGCCTTGGCTAGCTCTTCTGAATCGGGCATGGATTTCC-3′). This plasmid was then modified by inserting PCR products encoding either IMX313 creating the plasmid pIMX866 using the forward primer IMX1332 (5′-CTGATGTGTGCGGAGAGG-3′) and the reverse primer IMX1039 (5′-TAGAAGGCACAGTCGAGG-3′), or OVX313 creating the plasmid pIMX867 using the forward primer IMX1293 (5′-GGTCAGGATGATCAAACGTGGG-3′) and the reverse primer IMX1039 (5′-TAGAAGGCACAGTCGAGG-3′), from plasmids in laboratory stocks. Finally, an empty vector was constructed (pIMX719), by digestion of pGT-h-NP with the enzymes *Xba*I and *Hind*III, filling-in and re-ligating the ends, thus deleting the sequence encoding tPA and NP. All plasmids were verified by DNA sequencing. Two bacterial expression plasmids were used for overproducing the NP of the prototype human influenza isolate WS.^27^ A synthetic gene, codon optimized for expression in *Escherichia coli* and encoding OVX836, was purchased from DNA2.0 (now ATUMBIO) in the bacterial expression vector pDR441-SR. This plasmid, called pOVX836, was modified to delete the sequence encoding OVX313, creating the expression plasmid for NP (strain A/Wilson-Smith/1933) called pNP.

### Protein production and characterization

Both the NP and the OVX836 proteins were produced by using the bacterial strain BL21. Briefly, when bacterial growth reached the logarithmic phase, 0.5 mM isopropyl 𝛽-D-1-thiogalactopyranoside (IPTG) was used to induce expression for 16 h at 25 °C. Recombinant NP and OVX836 proteins were purified by means of affinity chromatography on a heparin sepharose column. The mutated form of NP (mut-NP and mut-OVX836) was an E339A/R416A mutant NP that did not self-associate and formed only monomers^29,59^ (Supplementary Figure 1b).

### SDS-PAGE analysis

Protein purity was evaluated by SDS-PAGE. Recombinant proteins were analyzed using a Criterion 4–12% Bis-tris gel (Bio-Rad), following the supplier's instructions. Briefly, proteins were denatured and reduced using NuPAGE LDS sample buffer (4×, Thermo) and NuPAGE reducing agent (10×, Thermo), respectively. The samples were heated at 70 °C for 10 min and then 0.5 µg of proteins were loaded on the Criterion 4–12% Bis-tris gel. The electrophoretic separation was carried out using a MOPS SDS running buffer at a constant voltage (200 V) for 50 min, using the NuPAGE antioxidant (Life Technologies). Proteins were detected using Instant Blue (Euromedex). Precision Plus Protein Standards (Bio-Rad) were used as molecular weight marker. Gel images were acquired with a Chemidoc Touch Imager (Bio-Rad) and images were then analyzed using the Image-Lab software (Bio-Rad).

### Western blot

Western blotting, using an antibody directed against NP antigen (MAb anti-NP, Clone 7B4G10G8), was performed to establish the identities of OVX836 and NP. Recombinant proteins were denatured and reduced as described previously. 1D-PAGE was performed as described above. The gel was then blotted onto a nitrocellulose membrane using a Trans-blot transfer system and a Trans-blot nitrocellulose pack (Bio-Rad) following the supplier's instructions. Precision Plus Protein Western C Standards (Bio-Rad) were used as molecular weight markers. The membrane was blocked with 3% bovine serum albumin protein (BSA) in Tris-buffered saline-Tween-20 (TBS-T) (0.1%T, w/v) and then probed with the monoclonal antibody directed against NP antigen (MAb anti-NP, 1/3000). Peroxidase-conjugated goat anti-mouse secondary antibodies were purchased from Thermo (1/30000), and the chemiluminescence detection kit (Clarity ECL Western blot substrate) from Bio-Rad. Western blot signals were acquired with an instrument for western blot imaging (Chemidoc Touch Imager, Bio-Rad) and images were analyzed using the Image-Lab software (Bio-Rad).

### Liquid chromatography

RP ultra-high-performance liquid chromatography (RP-UHPLC) was performed to provide information on protein homogeneity. Protein species were separated on a non-polar stationary phase with a gradient of hydrophobicity. Briefly, around 4.5 μg of protein were injected on a MAbPac RP column (Thermo, 4 μm, 2.1 × 100 mm), thermostated at 40 °C. The elution (0.4 mL/min) was performed using a linear gradient of acetonitrile with 0.1% Trifluoroacetic acid (TFA) and monitored by UV detection at 280 nm. The reducing agent TCEP (10 mM final) was added to reduce proteins for RP-UHPCUV analysis.

High performance size exclusion chromatography (SE-UHPLC-UV) was performed in order to analyze product quaternary structures and sizes of recombinant molecules. Briefly, a BEH SEC450 (Waters, 4.6 × 150 mm, guard 4.6 × 30 mm), equilibrated at 30 °C, was used in combination with 50 mM Na/K_2_ phosphate buffer pH 6.8, 0.5 M NaCl, 0.8 M arginine mobile phase. In all, 10 µg of protein sample was loaded onto the column and monitored by UV detection at 280 nm.

### Cell preparation and flow cytometry analysis

Flow cytometry and fluorescence-based imaging flow cytometry were used to analyze the uptake of proteins labeled with Alexa Fluor 647 fluorochrome (Molecular Probes, Invitrogen Life Technologies). Bone marrow DC were generated from C57BL/6 mice, cells were isolated by flushing femurs and tibias with culture medium RPMI-1640 (Invitrogen) containing 10% heat-inactivated Fetal Bovine Serum (FBS), 10 μg/ml gentamicin, 2 mM l-glutamine (Invitrogen), 50 μM 2-Mercaptoethanol (2-ME) (Sigma-Aldrich), and 10 mM HEPES (Invitrogen). Cells were filtrated through a 100-μm nylon cell strainer (BD Falcon), centrifuged, and cultured at 2 × 10^6^ cells/ml in medium supplemented with 100 ng/ml recombinant human Flt3l (Amgen).^61^ Flt3L-DC at days 7–8 of culture were incubated for the indicated time with Alexa647-labeled proteins. DC were washed and incubated 15 min with Live Dead reagent in PBS 1 × (Life technologies, 0.5 µl per well). DCs were collected and stained 45 min at 4 °C with antibodies specific for the murine cell surface antigens in FACS buffer (PBS1X, 1% FCS, 0.09% azide): CD11c (clone HL3), B220 (RA3-6B2), CD24 (M1/69), CD172a (P84) (all BD Biosciences). Prior to acquisition, cells were fixed in 2% paraformaldehyde. Acquisition was performed on LSR Fortessa flow cytometer (BD Biosciences) and post-collection data were analyzed using FlowJo software (TreeStar). For fluorescent imaging experiments, data and images were collected on an Amnis ImageStreamX Mark II, and at least 5000 events per sample were acquired. Data were analyzed using IDEAS software (EMD Millipore) and magnification of ×60 was used for all images shown.

For in vivo experiments, intravascular staining with anti-CD45-BV421 was performed 3 min before killing of the mice, thereby staining all intravascular, but not parenchymal, lymphocytes in order to identify lung resident memory CD8+ T cells as described.^62^ Mouse spleens and lungs were collected and dissociated by mechanical disruption with GentleMACS Dissociator (spleens or lungs) (Miltenyi Biotec). Single-cell suspensions were stained with antibodies against CD45 (30F11), CD8a (53.6), CD44 (IM7) (all BD Biosciences), and analyzed by flow cytometry. NP-specific CD8+ T cells in lungs and spleens were detected using pentamer staining, cells were incubated with Major Histocompatibility Complex (MHC) class I (H-2Db) pentamers specific for influenza virus NP_366–374_ - PE (H2Db-ASNENMETM) (Proimmune).

### Animals and immunizations

Six-week-old female C57BL/6 mice (Charles River Laboratories, Lyon, France) were used in all experiments, except for the challenge test, for which 6-week-old female Balb/c mice (Charles River Laboratories, Quebec, Canada) were also used as specified on each case. Animals were maintained under specific pathogen-free conditions, with ad libitum access to food and water. All animal procedures were approved by the Institutional Animal Care ethics committee of the Plateau de Biologie Expérimental de la Souris (CECCAPP_ENS_2015_004) and the Institutional Animal Care Committee of the Center Hospitalier Universitaire de Québec (CPAC #2013-134), in full accordance with European regulations and the guidelines of the Canadian Council on Animal Care, respectively.

DNA immunizations: to determine the effects of IMX313 and OVX313 on the immunogenicity of NP in plasmids, DNAs (50 µg) in endotoxin-free PBS were administered i.m. into the gastrocnemius muscle on days 0 and 14.

Immunogenicity and dose-response with recombinant proteins: to compare the immunogenicity of the OVX836 protein to that of NP, mice were i.m. immunized twice three weeks apart with 6.25, 12.5, 25, or 50 µg of the respective proteins.

Co-administration of OVX836 and the seasonal influenza vaccine: to determine whether the co-administration of OVX836 protein with IIV vaccines could potentially stimulate both highly specific T-cell responses to NP and increased antibody responses to hemagglutinins present in the seasonal vaccine, groups of female C57BL/6 mice were i.m. immunized twice, 21 days apart with 25 µg of OVX836, with or without IIV (Fluarix®, GSK 2014–2015 formulation, 1.5 µg of each HA in 50 µL). Immunizations were performed by injection into the gastrocnemius muscle or two different muscles (gastrocnemius and calf muscle) for double administration (OVX836 and IIV), with both injections being administered in the same hind limb.

Blood samples were collected before each immunization and 2 weeks or (14 days) after the booster immunization, mice were sacrificed and both serum, lung and splenocytes were collected from each mouse and processed individually.

### Mice challenge studies

Influenza challenge studies were conducted at the Infectious Disease Research Centre of the CHU de Quebec and Laval University (Quebec, Canada) and at the Plateau de Biologie Expérimental de la Souris (Lyon, France). Groups of 12 female C57BL/6 or Balb/c mice were immunized in two different muscles of the same hind limb with either 25 µg (in 25 µl) of the OVX836 protein, of the IIV (Fluviral®, GSK 2014–2015 or Influvac®, 1.5 µg of each HA in 50 µL), or a combination of both (OVX836/IIV). Two immunizations (21 days apart) were performed for each group, and a PBS-immunized group was included as control. Three weeks after the second immunization, mice were again lightly anesthetized by isoflurane and infected through an i.n. instillation with 20 µl of a viral suspension, containing either 3LD50 of influenza H1N1 A/California/7/2009, 1LD50 (in Balb/c mice) or 2LD50 (in C57BL/6 mice) of H1N1 A/WSN/33 or 1LD50 of H3N2 A/Victoria/5/72 strain, as indicated in each case. Of note, the H1N1 A/California/7/2009 strain is homologous to the H1N1 strain present in the IIV vaccine, whereas the other two strains used in this study are not. All mice were monitored daily for survival and weight loss during 14 days. Four mice per group were randomly chosen and sacrificed on day 4 (A/California/7/2009 and A/WSN/33) or 5 (A/Victoria/5/72) post-challenge, and their lungs were aseptically removed for the determination of LVTs by standard plaque assay in MDCK cells. Serum samples were collected from each animal before each immunization and viral challenge, as well as on day 21 post-challenge, to evaluate the specific IgG antibody response by HAI assays. Serologic tests were performed against the same virus strains used for the viral challenge, as well as the strains contained in the IIV vaccine (A/California/7/2009 (H1N1), A/Texas/50/2012, and B/Massachusetts/2/2012), following the standard WHO guidelines.^63^

### Ex vivo IFN-γ enzyme-linked immunoSpot (ELISpot) assay

Influenza NP-specific T cells secreting IFN-γ were enumerated using an IFN-γ ELISPOT assay (Mabtech). Lymphocytes were isolated from the spleen and the lung from individual mice. CD4+ T cells were purified by positive selection using a MACS Isolation Kit for mice with CD4 (L3T4). ELISpot plates were coated with the capture mAb (#3321-2 H) then incubated overnight at 4 °C according to the instruction manual of Mabtech. Then, 2 × 10^5^ T cells and CD4+ cells were cultured for 20 h at 37 °C/5% CO_2_ with 5 µg/mL of recombinant NP protein (OSIVAX). In addition, 2 × 10^5^ T cells were stimulated in similar conditions with 5 µg/mL of the NP_366-374_ (GenScript) immunodominant peptide epitope in C57BL/6 mice presented by H-2Db to stimulate CD8+ T cells.^64^ Concavalin A (Sigma) was used as a positive control and unstimulated splenocytes/lung cells were used as negative controls. Spots were counted with an ELISPOT reader system (CTL-ImmunoSpot® S6 Ultra-V). The number of protein- or peptide-reactive cells was represented as spot-forming cells (SFCs) per 2 × 105 cells per well.

### Cytokine production by ELISA after in vitro stimulation

For analysis of cytokine secretion, lung cells were cultured (2 × 10^6^ cells/ml) for 48 h with 5 µg/ml peptide NP_366-374_. Supernatants were collected and cytokine production was measured by sandwich ELISA kit (Mabtech) for IFN-γ (3511-1H), IL-2 (3311-1H), TNFα (3441-1H), and IL-4 (3321-1H) according to the protocols provided by the manufacturer. Cytokine concentrations in pg/ml were quantified using an appropriate standard curve.

### Antibody ELISA

Levels of IgG were measured in serum samples collected on days 35. The 96-well ELISA plates were pre-coated with 100 µL of recombinant NP (OSIVAX) at 5 µg/mL overnight at 4 °C. A total of 100 µl of serial twofold dilutions of sera from each group of immunized mice were added to each well and incubated for 2 h at 25 °C. Bound antibody was detected with goat anti-mouse IgG-HRP (Life Technology) and finally, 100 μl of tetramethylbenzidine (Interchim) substrate was added to each well. The antibody levels in serum were expressed as logarithm of endpoint dilution titer and this is defined as the reciprocal of the highest analytic dilution that gives a reading threefold over the mean Optical Density (O.D.) 650 value of the negative-control mice serum at the 1/100 dilution. A value of 1.70 (log) was arbitrarily chosen to illustrate a titer <100.

### Statistical analysis

The plotting of data and statistical analysis were performed using GraphPad Prism 7 software. Statistical significance was determined using the unpaired, one-way analysis of variance (ANOVA) with Tukey’s multiple comparisons test or non-parametric Kruskal–Wallis test followed by Dunn’s multiple comparisons test. Differences were considered significant if the *p*-value was *p* < 0.05. Survival rates of mice were compared using Kaplan–Meier survival analysis, and statistical significance was assessed using the log-rank (Mantel–Cox) test. For weight loss curves, groups were compared at each day post-infection using Kruskal–Wallis test followed by Dunn’s multiple comparison test.

### Reporting summary

Further information on experimental design is available in the Nature Research Reporting Summary linked to this article.

## Supplementary information

Supplementary Figure 1, Supplementary Figure 2, Supplementary Figure 3, Supplementary Table 1

Reporting Summary

## Data Availability

The data that support the findings of this study are available from the corresponding author upon reasonable request.

## Supplementary information

**Supplementary information** accompanies the paper on the *npj Vaccines* website (10.1038/s41541-019-0098-4).
