# Supplementary material for: OVX836 a recombinant nucleoprotein vaccine inducing cellular responses and protective efficacy against multiple influenza A subtypes
Source: NPJ Vaccines. 2019 Jan 23;4:4. doi: 10.1038/s41541-019-0098-4 (PMC6344521; doi:10.1038/s41541-019-0098-4)
Supplement: Supplementary file 1 — Supplementary Figure 1, Supplementary Figure 2, Supplementary Figure 3, Supplementary Table 1 [file 41541_2019_98_MOESM1_ESM.pdf]

OVX836 a recombinant nucleoprotein vaccine inducing cellular responses and protective efficacy against multiple influenza A subtypes

Judith del Campo[1]*, Andres Pizzorno[2 §], Sophia Djebali[3 §], Julien Bouley[1], Marjorie Haller[1], Jimena Pérez-Vargas[1,6], Bruno Lina[2,4], Guy Boivin[5], Marie-Eve Hamelin[5], Florence Nicolas[1], Alexandre Le Vert[1], Yann Leverrier[3], Manuel Rosa-Calatrava[2], Jacqueline Marvel[3], Fergal Hill[1] *.

Supplementary Files

a

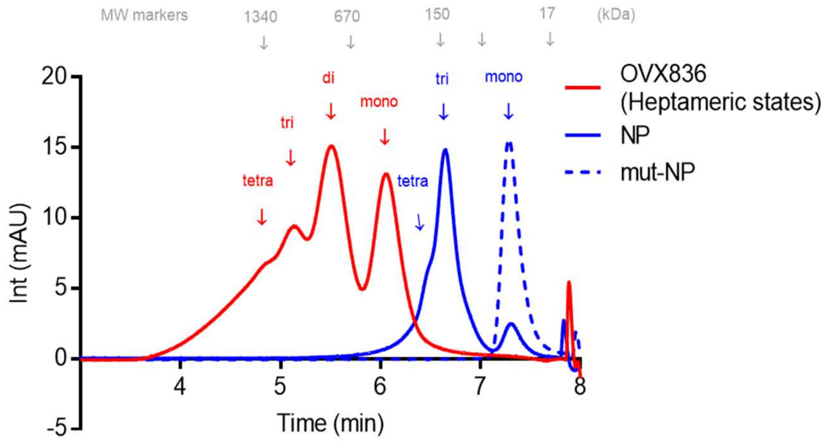

MW markers
1340
670
150
17
(kDa)
Int (mAU)
Time (min)
tetra
tri
di
mono
tri
mono
tetra
OVX836
(Heptameric states)
NP
mut-NP

b

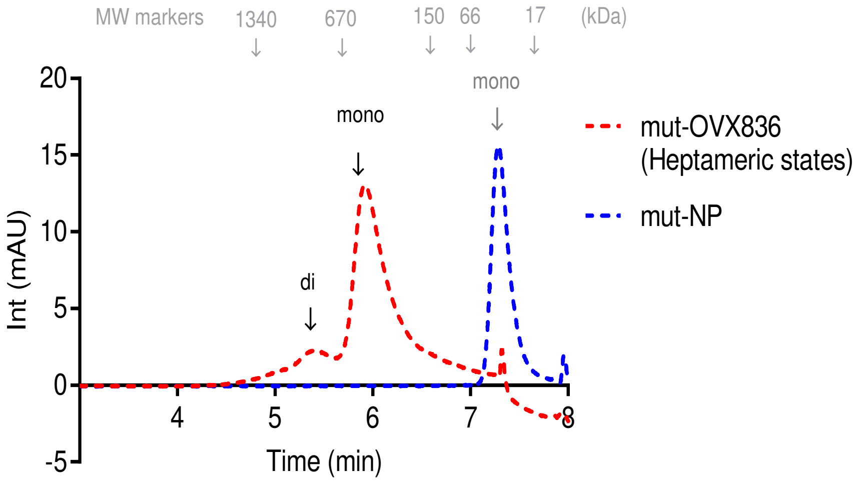

MW markers
1340
670
150
66
17
(kDa)
mono
mono
di
Int (mAU)
Time (min)
mut-OVX836
(Heptameric states)
mut-NP

c

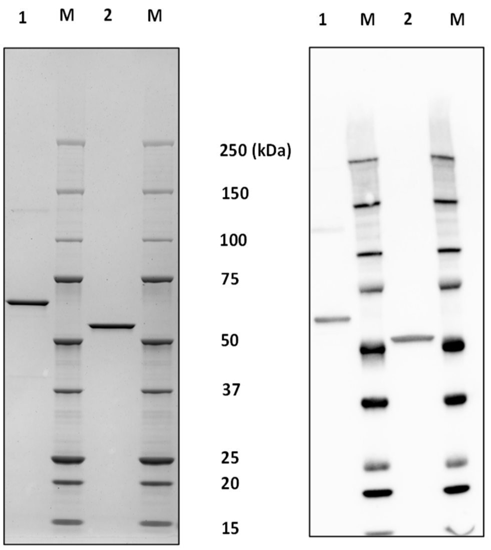

1
M
2
M
250 (kDa)
150
100
75
50
37
25
20
15

Supplementary Figure 1. OVX836 protein is an oligomeric form of NP

(a) Gel filtration profiles of OVX836 and NP proteins. Quaternary structures were evaluated by gel filtration chromatography. Monomeric (mono), dimeric (di), trimeric (tri) and tetrameric (tetra) states. OVX836 molecules are mainly organized as mono, di- and tri-heptamers (400-1200 kDa). Wild-type NP molecules are mainly organized as trimers (150 kDa). The E339A/R416A-mutated NP (mut-NP), as expected, no longer formed small oligomers, with a main peak corresponding to a monomer of NP (between 17 and 66 kDa). (b) Gel Filtration profile of mut-OVX836 and mut-NP. Quaternary structures were evaluated by gel filtration chromatography. Monomeric (mono) and dimeric (di) states. Note: this OVX836 tail loop mutant not only forms heptamers but also forms a species at the ~900-kDa position, which corresponds to the molecular mass of a mut-OVX836 dimer. This formation of homodimers indicates that mut-OVX836 is able to dimerize through an interface that does not involve the tail loop, as observed for the native NP protein [29]. (c) Representative SDS-PAGE analysis of OVX836 and NP proteins. Polypeptides were fractionated on 4-12 % Bis-tris gels and stained with Instant Blue, total protein were stained (left panel) or blotted onto a membrane and probed with anti-NP Mab (right panel). OVX836 and NP migrated at 64 and 56 kDa, respectively (lanes 1 and 2). Molecular weight (MW) markers (lanes M). The SDS-PAGE and the Western-blot derive from the same experiment and were processed in the same day.

a

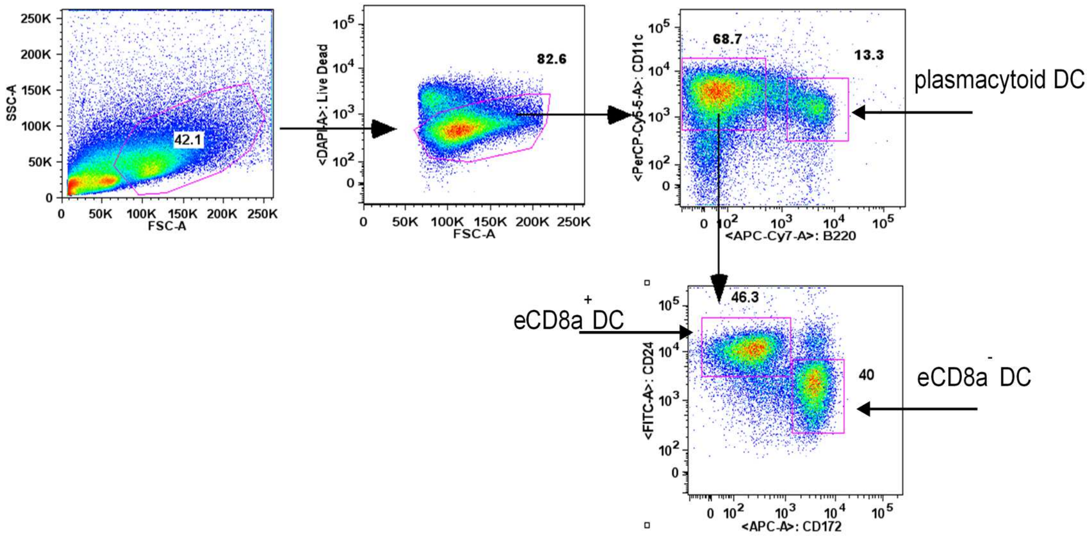

b

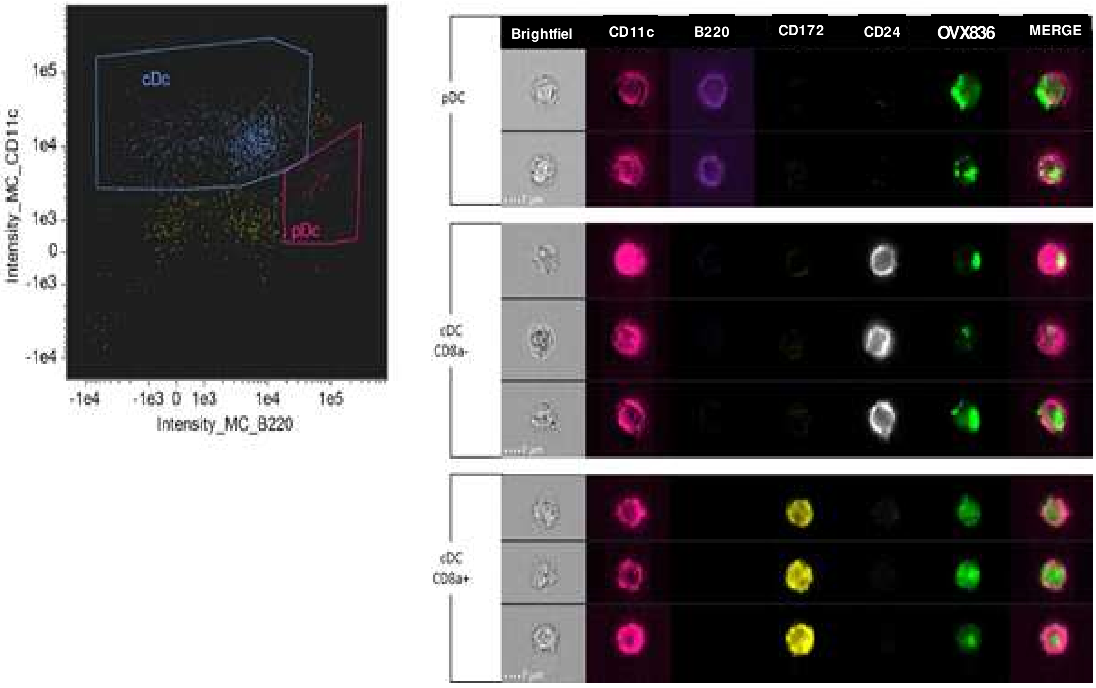

Supplementary Figure 2. Incorporation of OVX836-Alexa647 by Flt3L-DC

(a) Gating strategy to identify the different DC populations: pDC (CD11c+ B220+) and cDC (CD11c+ B220-). cDC can be further subdivided into eCD8α- (CD24- CD172+) and eCD8α+ (CD24+ CD172-) subsets. (b) Imaging flow cytometry was used to visualize OVX836-Alexa647 incorporation by Flt3L-DC derived from C57BL/6 mice. Flt3L-DC were incubated

16 hours with NP or OVX836-Alexa647 (50µg/ml) at 37°C and surface stained for CD11c, B220, CD24 and CD172a. Representative cells for each DC subset (pDC: CD11c+ B220+; cDC eCD8α-: CD11c+ B220- CD24Lo CD172a+; cDC eCD8α+: CD11c+ B220- CD24Hi CD172a) are shown, from two independent experiments. Original magnification ×60.

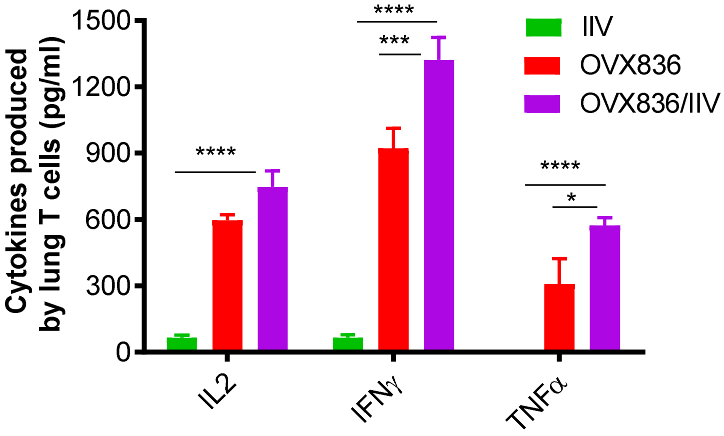

Supplementary Figure 3. Cytokines produced by OVX836 in lung T cells

Production of IL-2, IFN-γ, TNF-α assessed by ELISA. Supernatants were collected 48h after NP366-374 peptide stimulation. The results represent the mean with ±SD for five mice per group were shown as bars of each of the three cytokines. Differences were assessing by 1-way ANOVA followed by Tukey's Multiple comparison test with 95% confidence intervals; $p<0.05$ is considered significant (*$p<0.05$, ***$p<0.001$, ****$p<0.0001$).

Supplementary Table 1. Hemagglutination Inhibition (HAI) titers. Serologic tests against the virus strains used for the viral challenge, as well as the strain contained in the IIV vaccine

HAI titers were measured in pooled serum samples (2 pools per group) before (Pre) and after (Post) challenge and expressed as the reciprocal of the limit dilution. Serologic tests were performed against the same virus strains used for the viral challenge influenza H1N1 A/California/7/2009 or H3N2 A/Victoria/5/72 virus, as well as the H3N2 A/Texas/50/2012 strain contained in the IIV vaccine.

			Viral strain used for HAI assay					
			A/California/07/2009		A/Texas/54/2012		A/Victoria/3/75	
			Pre	Post	Pre	Post	Pre	Post
A/California/07/2009*	PBS	Pool 1	<10	x	<10	x	<10	x
		Pool 2	<10	x	<10	x	<10	x
	OVX836	Pool 1	<10	80	<10	<10	<10	<10
		Pool 2	<10	40	<10	<10	<10	<10
	IIV	Pool 1	20	160	40	80	<10	<10
		Pool 2	10	80	20	40	<10	<10
	OVX836/ IIV	Pool 1	80	320	80	160	<10	<10
		Pool 2	80	320	80	80	<10	<10
A/Victoria/3/75*	PBS	Pool 1	<10	<10**	<10	10**	<10	320**
		Pool 2	<10		<10		<10	
	OVX836	Pool 1	<10	<10	<10	20	<10	320
		Pool 2	<10	<10	<10	10	<10	160
	IIV	Pool 1	10	<10	160	640	<10	640
		Pool 2	10	20	80	320	<10	640
	OVX836/ IIV	Pool 1	40	20	40	320	<10	160
		Pool 2	40	40	160	>1280	<10	160

* Strain used for viral challenge

** Results from one remaining mouse
